# TRNT1 deficiency: clinical, biochemical and molecular genetic features

**DOI:** 10.1186/s13023-016-0477-0

**Published:** 2016-07-02

**Authors:** Yehani Wedatilake, Rojeen Niazi, Elisa Fassone, Christopher A. Powell, Sarah Pearce, Vincent Plagnol, José W. Saldanha, Robert Kleta, W Kling Chong, Emma Footitt, Philippa B. Mills, Jan-Willem Taanman, Michal Minczuk, Peter T. Clayton, Shamima Rahman

**Affiliations:** Genetics and Genomic Medicine Programme, UCL Institute of Child Health, London, UK; MRC Mitochondrial Biology Unit, Cambridge, UK; UCL Genetics Institute, London, UK; Division of Mathematical Biology, National Institute for Medical Research, Mill Hill, London, UK; Division of Medicine, UCL, London, UK; Radiology Department, Great Ormond Street Hospital, London, UK; Metabolic medicine department, Great Ormond Street Hospital, London, UK; Department of Clinical Neurosciences, UCL Institute of Neurology, London, UK; Mitochondrial Research Group, Genetics and Genomic Medicine Programme, UCL Institute of Child Health, 30, Guilford Street, London, WC1N 1EH UK

**Keywords:** Mitochondrial disease, TRNT1, tRNA, Protein translation, SIFD, Sideroblastic anaemia

## Abstract

**Background:**

TRNT1 (CCA-adding transfer RNA nucleotidyl transferase) enzyme deficiency is a new metabolic disease caused by defective post-transcriptional modification of mitochondrial and cytosolic transfer RNAs (tRNAs).

**Results:**

We investigated four patients from two families with infantile-onset cyclical, aseptic febrile episodes with vomiting and diarrhoea, global electrolyte imbalance during these episodes, sideroblastic anaemia, B lymphocyte immunodeficiency, retinitis pigmentosa, hepatosplenomegaly, exocrine pancreatic insufficiency and renal tubulopathy. Other clinical features found in children include sensorineural deafness, cerebellar atrophy, brittle hair, partial villous atrophy and nephrocalcinosis.

Whole exome sequencing and bioinformatic filtering were utilised to identify recessive compound heterozygous *TRNT1* mutations (missense mutation c.668T>C, p.Ile223Thr and a novel splice mutation c.342+5G>T) segregating with disease in the first family. The second family was found to have a homozygous *TRNT1* mutation (c.569G>T), p.Arg190Ile, (previously published).

We found normal mitochondrial translation products using passage matched controls and functional perturbation of 3’ CCA addition to mitochondrial tRNAs (tRNA^Cys^, tRNA^LeuUUR^ and tRNA^His^) in fibroblasts from two patients, demonstrating a pathomechanism affecting the CCA addition to mt-tRNAs. Acute management of these patients included transfusion for anaemia, fluid and electrolyte replacement and immunoglobulin therapy. We also describe three-year follow-up findings after treatment by bone marrow transplantation in one patient, with resolution of fever and reversal of the abnormal metabolic profile.

**Conclusions:**

Our report highlights that *TRNT1* mutations cause a spectrum of disease ranging from a childhood-onset complex disease with manifestations in most organs to an adult-onset isolated retinitis pigmentosa presentation. Systematic review of all *TRNT1* cases and mutations reported to date revealed a distinctive phenotypic spectrum and metabolic and other investigative findings, which will facilitate rapid clinical recognition of future cases.

## Background

The field of monogenic mitochondrial disease has increased exponentially since the advent of next generation sequencing. Commencing with ‘targeted gene panels’ sequencing nuclear genes encompassing the mitochondrial proteome, moving rapidly to whole exome sequencing (WES) and more recently whole genome sequencing, it has enabled the diagnosis of many mitochondrial disease patients worldwide. New syndromes have been revealed [1, 2] and older syndromes such as Sengers syndrome have acquired genetic redefinition [3]. One of the processes that has been particularly associated with disease is mitochondrial translation. A number of new genetic disorders have recently been found in this pathway [4–7] with the majority of the newly reported cases being tRNA aminoacyl synthetase deficiencies. Furthermore the genetic disorders of mitochondrial protein synthesis have been flagged as an exciting area for clinicians as there appear to be tissue specific diseases making it possible to draw genotype-phenotype correlations [8].

In this study we explore a syndrome resulting from the deficiency of TRNT1 (CCA-adding tRNA nucleotidyl transferase enzyme), which performs an essential post-transcriptional modification by adding on the cytosine-cytosine-adenine (CCA) trinucleotide sequence to the 3′ end of all newly produced tRNAs. This TRNT1-dependent tRNA modification is essential for both cytosolic and mitochondrial tRNAs (mt-tRNAs) to participate in protein biosynthesis. The CCA trinucleotide sequence is required to accurately attach amino acids, to position the tRNA on the ribosome and also to conclude protein translation [9].

Recently, two different TRNT1 related disease entities have been reported; a haematological description of a congenital sideroblastic anaemia associated with immunodeficiency, fevers, and developmental delay (SIFD) in childhood [10–12] and an adult presentation of retinitis pigmentosa with a focus on ophthalmological features [13]. In this report we describe four cases including a new family with two severely affected children whose presentation encompassed both sets of clinical features, and in whom we have identified a novel *TRNT1* mutation. We explored the functional significance of the *TRNT1* mutations in these children and found clear evidence of impaired post-transcriptional modification of mt-tRNAs. We systematically review all the features reported in the cases published thus far and study the complete clinical phenotype, including a metabolic description that will enable physicians to diagnose this disease clinically and perform targeted genetic investigations.

## Methods

### Patients

We report one new family and also add a metabolic context to the phenotype of one previously reported family [10, 11]. Both families attended the metabolic clinic at our institution for diagnosis and treatment.

P1 was born to unrelated white European parents after a normal pregnancy. She presented at 2 weeks of age with a febrile illness associated with poor feeding and diarrhoea. No infective cause was found and she recovered from this episode. However over the next 3 years she had multiple recurring acute systemic illnesses. These episodes were characterised by fever, diarrhoea and vomiting, metabolic acidosis, electrolyte imbalance (episodic hyponatraemia, hypokalaemia, hypocalcaemia, hypomagnesaemia and hypophosphataemia), elevated hepatic transaminases and raised inflammatory markers.

She was investigated for an immune deficiency and was found to have severe hypogammaglobulinaemia with B cell maturation arrest requiring three-weekly sandoglobulin supplementation. She was also found to have a transfusion dependent sideroblastic anaemia with variable neutropaenia and thrombocytopaenia. Immunological investigations revealed low IgG, IgA and IgM (Table 1) while blood films showed anisocytosis, neutropaenia, pencil cells and elliptocytes. Bone marrow examination showed that most erythroblasts had abnormal siderosis with around 2/3 being ring or crescent forms. Megakaryopoiesis was abundant but with dysplastic nuclear morphology.

**Table 1:** TRNT1 deficient patients- biochemical and other laboratory data

Analyte	P1	P2	P3	P4	P4: 2 years post bone marrow transplant	Reference range
Plasma Lactate mmol/L	>2.5	3.1	1.2–2.8	1.4–2.7	1.1	<2
CSF Lactate mmol/L	2.5	NA	NA	NA	NA	<2
CSF protein g/L	0.41–1.79	NA	NA	NA	NA	<0.3
Plasma glycine μmol/L	504	248	280–332	310–344	235	100–330
Plasma threonine μmol/L	256	305	168–266	329–556	260	70–220
Plasma proline μmol/L	246	255	291–452	304–414	249	85–290
Plasma leucine μmol/L	91	133	140–245	96–198	129	65–220
Plasma isoleucine μmol/L	46	73	63–118	69–114	68	26–100
Plasma valine μmol/L	142	216	244–404	197–323	255	90–300
Plasma alanine μmol/L	296	458	562–875	572–665	346	150–450
Plasma ornithine μmol/L	101	107	83–210	112–241	145	25–120
White Cell Ubiquinone pmol/mg protein	NA	57.0	10.0	NA	NA	37–133
Creatine kinase u/L	NA	87–383	88	41–137	NA	75–230
Electrolytes mmol/L	Episodic hyponatraemia (131), hypokalaemia (2.7), hypocalcaemia (1.7), hypomagnesaemia (0.51), hypophosphataemia (0.89)	Normal	Episodic hyponatraemia, hypokalaemia, hypocalcaemia, hypomagnesaemia, hypophosphatemia	Not done during acute episode of illness	Normal	
Intact PTH pmol/L	3.3	NA	8.0	NA	NA	1.1–5.4
Gamma GT u/L	300	normal	NA	NA	NA	10–20
Urine organic acids	Mildly raised 3OH-butyrate with moderately raised adipate, suberate and 3OH-sebacate and mildly raised sebacate, C8:1 and C10:1 dicarboxylates, 3OH (C14:0, C8:1, C10:1) dicarboxylates	No abnormality	Strongly raised 3-hydroxybutyrate and acetoacetate	Mildly raised pyruvate	NA	NA
Urine amino acids	NA	NA	Generalised aminoaciduria	NA	NA	NA
Urine NAG/creatinine	612–2606	217	666	NA	NA	3.5–27.3
Urine RBP/creatinine	4531–25714	961	419	NA	NA	1.9–42.6
Hb g/L	Lowest 79 (freq transfusions)	66, 67	65–74	71, 68	(sample clotted)	114–145
Blood film	Anisocytosis, neutropaenia, dimorphic red cells, pencil cells, elliptocytes on film	Marked anisopoikilocytosis with hypochromic red cells, elliptocytes and fragments	Marked red cell anisopoikilocytosis. Many hypochromic, microcytic red cells and elliptocytes. Numerous red cell fragments. Occasional target cells	Neutrophils show toxic granulation marked RBC abnormalities: poikilocytosis, microcytosis, eliptocytosis, hypochromic red cells. polychromasia	NA	
IgG g/L	2.24–5.42 (3.7–15.8)	0.86 (3–10.9)	3.95, 10.6 (5.4–16.1)	4.11–4.32 (4.9–16.1)	10.90	
IgA g/L	<0.03 (0.3–1.3)	<0.06 (0.2–0.7)	0.15–0.62 (0.5–1.8)	0.17–0.41 (0.4–2.0)	0.76	
IgM g/L	0.11–0.23 (0.5–2.2)	0.17 (0.6–2.1)	0.09, 0.20 (0.5–2.2)	0.29–1.19 (0.5–2.0)	0.58	
Lymphocyte Subsets	Absent B cells	Low B cells	Low B cells	NA	NA	
Other	Moderate exocrine pancreatic insufficiency (stool elastase 182 μg/g, normal >200); later undetectable. Hair shafts: normal histology. Duodenal biopsy: partial villous atrophy. Liver iron:143 μg/100 ml dry weight. Normal very long chain fatty acids (VLCFA)	Pancreatic elastase 1 (μg/g) <15 Undetectable	Normal ammonia, blood spot carnitine profile, VLCFA, copper, caeruloplasmin, plasma methylmalonic acid (MMA), autoimmune profile; large bowel histology – mild patchy increase in eosinophils in lamina propria	Normal transferrin glycoforms, blood spot carnitine profile, VLCFA, urate, copper, caeruloplasmin, plasma MMA	NA	
Muscle histology	No ragged-red fibres or COX negative fibres	ND	NA	ND	NA	
Muscle respiratory chain enzymes (ratio to citrate synthase)	Complex I 0.139 Complex II + III 0.064 Complex IV 0.015	NA	Complex I 0.228 Complex II + III 0.068 Complex IV 0.007	NA	NA	Complex I 0.104–0.268 Complex II and III 0.040–0.204 Complex IV 0.014–0.034

NA: not available

She had recurrent episodes of encephalopathy associated with nystagmus and photophobia which appeared to improve markedly with benzodiazepine therapy. Development was globally delayed and she had retinal dystrophy. Brain MRI was initially normal but subsequently progressive cerebellar atrophy was observed. In addition, in the last stages of her disease, brain MRI showed widespread abnormalities with multiple lesions resembling infection. She developed recurrent bacterial infections and gastrointestinal involvement with chronic gastritis, partial villous atrophy, hepatosplenomegaly and pancreatic insufficiency (undetectable stool elastase).

Biochemical investigations revealed mildly elevated blood lactate at 2.5 mmol/L (reference range <2) and muscle mitochondrial respiratory chain complex IV activity at the lower end of the reference range (ratio to citrate synthase 0.015, reference range 0.014–0.034). Muscle histology was normal. In particular, neither ragged-red nor cytochrome oxidase-negative fibres were observed. She died at 3 years and 3 months following a progressive encephalopathy.

**Fig. 1:**
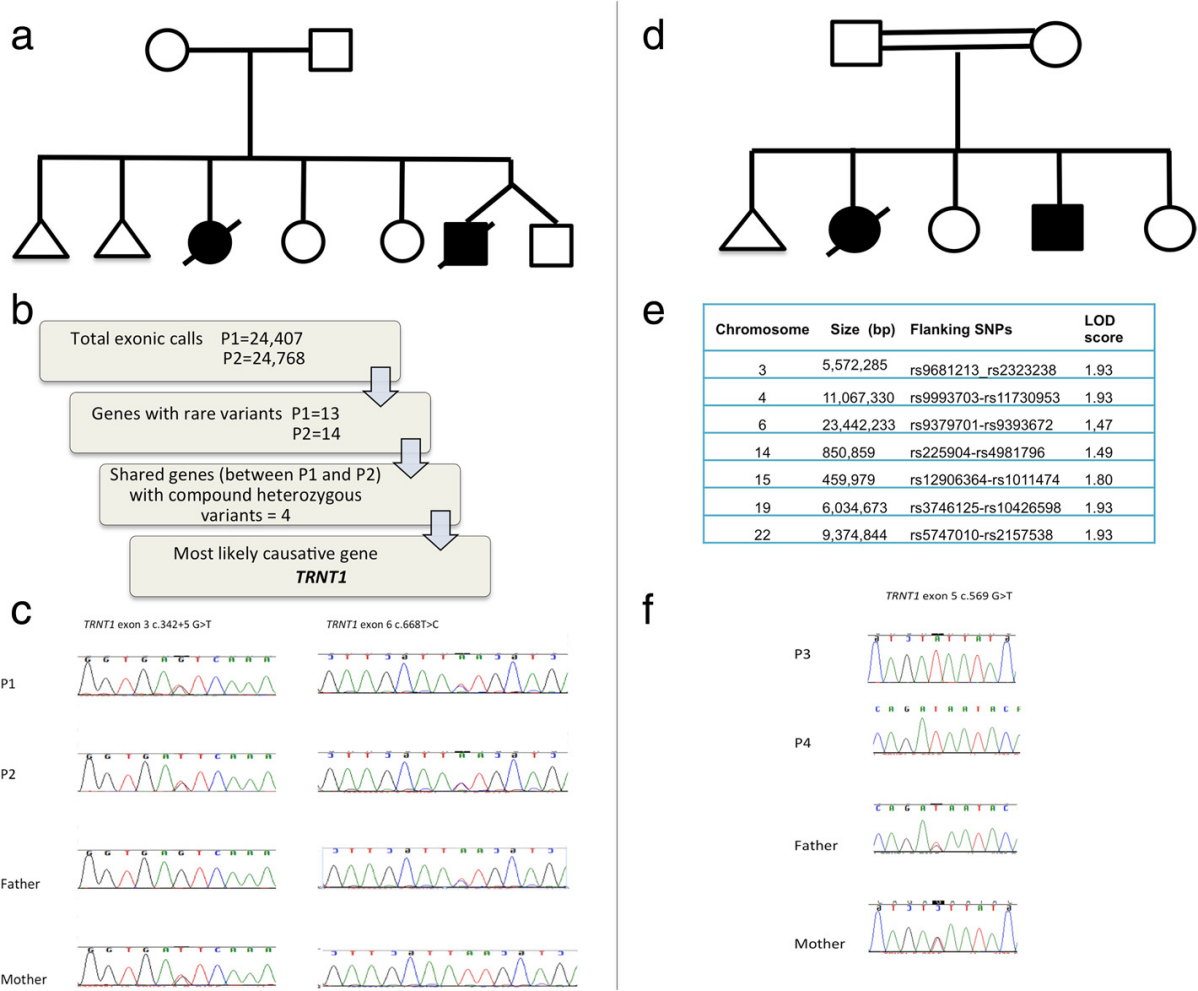
**a** Pedigree of family of P1 and P2. **b** Bioinformatic analysis of whole exome data. **c** Sanger sequencing results showing that P1 and P2 were compound heterozygous for the *TRNT1* mutations. **d** Pedigree of family of P3 and P4. **e** Linkage mapping data showing sizes of the chromosomal regions with the highest LOD scores. **f** Electropherogram demonstrating the *TRNT1* mutation in P3 and P4 (c.569G>T p.Arg190Ile) [11]

**Fig. 2:**
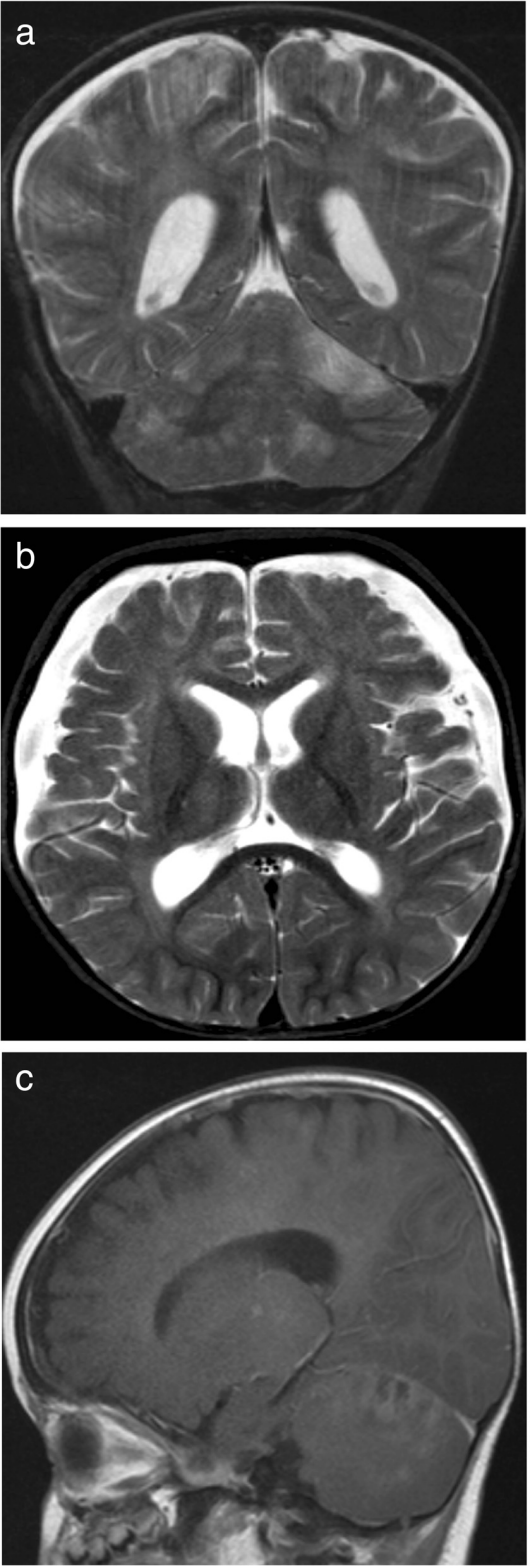
Axial (**a**) and Coronal (**b**) T2-weighted images and Sagittal (**c**) Post-contrast T1 weighted images of the brain MRI in P2, showing multiple focal lesions in the cerebral hemispheres and cerebellum involving both grey and white matter with contrast enhancement. Bilateral subdural collections were also present

P2 is the younger male sibling of P1 and is the older sibling of non-identical twins (Fig. 1a). He was admitted at 3 weeks of age with poor feeding, loose stools and pallor, and again at 4 weeks with poor feeding, fever and anaemia (haemoglobin 66 g/L). Although his blood film showed occasional nucleated red cells with possible sideroblastic granules, bone marrow examination at one month showed dyserythropoeisis but no ringed sideroblasts. He had central hypotonia, retinal pigmentation, hepatosplenomegaly and exocrine pancreatic insufficiency. Immunological investigations revealed initially low B cells which normalised and low IgG, IgA and IgM (Table 1). He had recurrent seizures and his MRI brain at 10 months demonstrated multiple focal lesions in the cerebral hemispheres and cerebellum (Fig. 2). He died at 10 months of age because of intractable status epilepticus.

P3 and P4, previously reported as cases 1 and 2 [10] were born to first cousin Pakistani parents (Fig. 1d). They had five children including a stillborn baby at 32 weeks’ gestation and two other daughters who are well except for radioulnar fusion due to Cenani-Lenz syndrome caused by a homozygous *LRP4* mutation [14]. P3 was first diagnosed with sideroblastic anaemia at 8 months. She developed recurrent episodes of fever from 1 year and global developmental delay, retinitis pigmentosa and bilateral sensorineural deafness. She had low B cells with hypogammaglobulinaemia and features of sideroblastic anaemia on bone marrow examination. She required numerous blood transfusions to maintain her Hb above 70 g/L. During the febrile episodes she developed hyponatraemia, hypokalaemia, hypocalcaemia, hypomagnesaemia and hypophosphatemia and generalised aminoaciduria. Plasma amino acid analysis revealed elevated proline 347 μmol/L (85–290), alanine 749 μmol/L (150–450) and glutamine 1014 μmol/L (480–800). Urine organic acids showed strongly raised 3-hydroxybutyrate and acetoacetate. Urate was mildly raised and red cell nucleotide profile showed the presence of some abnormal nucleotides. At four years, she was found to have a urate renal calculus which resulted in right sided hydronephrosis. Porphyria screen and sequencing of exons 9 and 11 of the mevalonate kinase gene (performed because of the recurrent fevers) were normal. Muscle biopsy demonstrated markedly reduced complex IV activity expressed as a ratio to citrate synthase at 0.007 (0.014–0.034) (Table 1). She died after multiorgan failure and presumed cephalosporin-induced toxic epidermal necrolysis at 14 years.

Her younger brother P4 had global developmental delay and hypotonia with a microcytic anaemia. He presented at two months with high fever, vomiting and diarrhoea. Anaemia (haemoglobin 71 g/L) and panhypogammaglobulinaemia were noted (Table 1). His blood film showed slightly immature neutrophils with toxic granulation and marked red cell abnormalities including poikilocytosis, microcytosis, elliptocytosis, hypochromic red cells and polychromasia, similar to the findings in his sister P3. He had fever recurring every 7–10 days, which was associated with vomiting and diarrhoea. Urine organic acids revealed mildly raised pyruvate. Pearson syndrome was excluded in both P3 and P4 by screening for mitochondrial DNA deletions. P4 underwent bone marrow transplantation (BMT) after which his fevers resolved, his growth improved and he made some developmental progress. He remains systemically well 3 years post BMT. At 10.5 years of age he has started to walk independently and is making progress with speech, but continues to have moderate hearing loss and retinopathy.

### Whole exome sequencing

Genetic investigations were performed with ethical approval from the National Research Ethics Committee London Bloomsbury, UK and informed consent was obtained from parents for investigations and publication of findings. Whole exome sequencing (WES) was performed in P1 and P2 using the Agilent SureSelect capture kit and Illumina’s HiSeq 2000 high throughput sequencing. Raw fastq files were aligned to the hg19 reference genome using novoalign version 2.08.03. We processed 2600 in-house control samples together with these samples to create gVCF files using the Haplotype Caller module of the Genome Analysis Tool Kit (GATK) version 3.1.1. These individual gVCF files were pooled into combined gVCF of 100 samples, and used for variant calling (Genotype gVCF module of GATK 3.1.1). Variant quality scores were re-calibrated according to GATK recommendations for indels and SNPs. Rare variants (defined as an allele frequency <0.5 % in our in-house control samples) which were splicing, nonsynonymous, frameshift or presumed loss-of-function and present in both P3 and P4 were prioritised. In view of non-consanguinity, compound heterozygous inheritance was assumed.

### Homozygosity mapping

Whole genome-wide SNP genotyping was performed in P3 and P4, their parents and one unaffected sibling using the Illumina HumanCytoSNP-12 microarray. LOD scores were calculated as published before [15]. All processing was carried out in accordance with the Infinium HD Ultra Assay protocol (Rev B, 2010, Illumina Inc, San Diego, USA). Data were initially analysed assuming an autosomal recessive model using the Illumina Genome Studio software which generates genotypes, copy number and loss of heterozygosity data (cnvPartition v3.1.6, Illumina). Quality control checks were performed to assess data quality. Samples were assessed for their call rate which should be >98 and >99 % average across the batch. The B-allele frequency plots and copy number analysis results were checked to identify potentially contaminated samples.

### Sanger sequencing of mutations

All exons and exon-intron boundaries of candidate genes located within homozygous intervals in P3 and P4 were amplified (GoTaq PCR Kit Promega) according to the manufacturer’s protocol and Sanger sequenced. The *TRNT1* mutations identified by WES in P1 and P2 were also confirmed by Sanger sequencing, using primer sequences for *TRNT1* exons 3 and 6 (exon 3: Forward ATAGCAGGAGGAGCAGTGAG and reverse GACTGCAGGGTTTATGACGG and exon 6 forward CAGATTTTGCTTGTGATATGCCA and reverse ACCCCATAACCCAAACTTTGTC) All PCR products were sequenced using the Big Dye Terminator Reaction Kit on a 2400GeneAmp® 9700 sequencer. Sequencing data were analysed using the Sequencher v. 5.2.4 software.

### Western blot

Western blot was performed as described previously [16] using OXPHOS antibody cocktail (Abcam) in P1 and P2 compared to controls. Fibroblast cells were not available for P3 and P4.

### Mitochondrial translation assay

Patient and control fibroblasts were plated on 6-well plates. At 80 % confluence, they were washed twice with methionine and cysteine-free DMEM (1 mL/well of a 6 well plate). Labelling medium (600 μL) was added to each well and incubated for 20 min at 37 °C, before adding emetine (SIGMA, 25 mg/ml in PBS) to a final concentration of 100 μg/mL with a further incubation at 37 °C for 30 min. 100 μCi L-[35S]-methionine (Perkin Elmer - NEG009L005MC) was then added to each well to a final concentration of 166.6 μCi/mL per well and incubated for 30 min at 37 °C. The cells were washed with PBS and trypsinised, protein was isolated and concentration determined using BCA assay, and western blot was performed. The gel was stained with SimplyBlue SafeStain (Thermo Fisher Scientific), dried at 80 °C under vacuum for 1 h and exposed to a phosphorimage screen for 72 h.

### Analysis of mitochondrial tRNA CCA sequences by circularisation reverse transcription–PCR assay

Circularisation reverse transcription PCR (cRT–PCR) and sequencing were used to determine both 5’ and 3’ ends of mt-tRNA as described previously [17]. Briefly, 2.5 μg of total RNA was circularized with T4 RNA ligase (New England Biolabs) in the presence of DNase I (Roche) followed by phenol–chloroform extraction and ethanol precipitation. Reverse transcription was carried out using Omniscript Reverse Transcriptase (Qiagen) with a gene-specific reverse primer (tRNA_His_R1).

Newly synthesized cDNA was used as a template for a PCR reaction, primed with R1 primer and a forward primer, F1. The region amplified contained the junction of the 5’ and 3’ extremities. Products were cloned using TOPO TA Cloning Kit (Life Technologies) and sequenced. The following primers were used in the analysis: tRNA_His_Reverse: CAATCTGATGTTTTGGTTAAACTATA, tRNA_His_Forward: AATCTGACAACAGAGGCTTACGACCC, tRNA_SerAGY_Reverse: CATGAGTTAGCAGTTCTTGTGAGC TT, tRNA_SerAGY_Forward: CCCCATGTCTAACAACATGGCTTTC, tRNA_Cys_Reverse: TGCAATTCAATATGAAAATCACCTCG and tRNA_Cys_Forward: TTCGAAGAAGCAGCTTCAAACCTGCC. The following TOPO clones were sequenced: (a) mt-tRNA^His^ C1 29 clones, P1 32 clones, P2 29 clones (b) mt-tRNA^Cys^ 16 clones for C1, P1 and P2 (c) mt-tRNA^SerAGY^ C1 13 clones, P1 16 clones, P2 15 clones.

### Northern blotting

Northern blot analysis was performed as described previously [18]. Briefly, total RNA was isolated from primary skin fibroblasts using TRIzol® LS Reagent (Life Technologies) according to the manufacturer’s instructions. RNA was resolved by Urea-PAGE on a 10 % gel, transferred to a nylon membrane, UV-crosslinked to the membrane and hybridised with radioactively labelled T7-transcribed RNA probes.

### Protein modelling

The molecular graphics program MOE (Molecular Operating Environment, 2012) running on an Ubuntu Linux workstation was used to visualize, mutate and analyse the crystal structure of full-length human mitochondrial CCA-adding enzyme [19] solved at a resolution of 1.9A.

### Identification of other cases

A PubMed search was performed using the terms‛TRNT1’, ‘SIFD’ and ‘sideroblastic anaemia’. Since the gene *TRNT1* was only associated with human disease in 2014 we searched for publications from 2014 to date. Only papers reporting cases with confirmed *TRNT1* mutations were included.

## Results

### Genetic findings

#### Exome sequencing

Only four genes (*CEL, SLC12A1, SRRM, TRNT1*) containing compound heterozygous mutations shared between the affected siblings P1 and P2, were found after bioinformatic filtering (Fig. 1b). Of these, *TRNT1* appeared the most likely causative gene given the clinical history. We identified a novel splice mutation c.342+5G>T together with a known pathogenic missense mutation, c.668T>C p.Ile223Thr. Parents were each heterozygous for one mutation, confirming segregation with disease in this family (Fig. 1c).

#### Homozygosity mapping

Homozygosity mapping was performed prior to the advent of WES in P3, P4, parents and an unaffected sibling. The mapping revealed 7 significant homozygous regions shared between the two affected siblings spanning a total of 56.8 megabases, including four regions with a LOD score of 1.93 (Fig. 1e) containing 716 genes including 11 genes with a Maestro (predicted mitochondrial localisation) score >10 (*TRNT1*, *GRPEL1*, *LONP1*, *NDUFA11*, *CLPP*, *TIMM44*, *NDUFA7*, *ATP6V1E1*, *SLC25A1*, *TXNRD2* and *CHCHD10*). The *TRNT1* mutation illustrated in Fig. 1f (c.569G>T, p. Arg190Ile) in this family has previously been published by another group [11] who were also investigating this family, unbeknown to us.

### Western blot

Immunoblotting showed no decrease in the steady-state levels of either mtDNA or nuclear encoded OXPHOS subunits in the patients (P1 and P2) when compared with controls.

### Mitochondrial translation and analysis of CCA addition to mitochondrial tRNAs

**Fig. 3:**
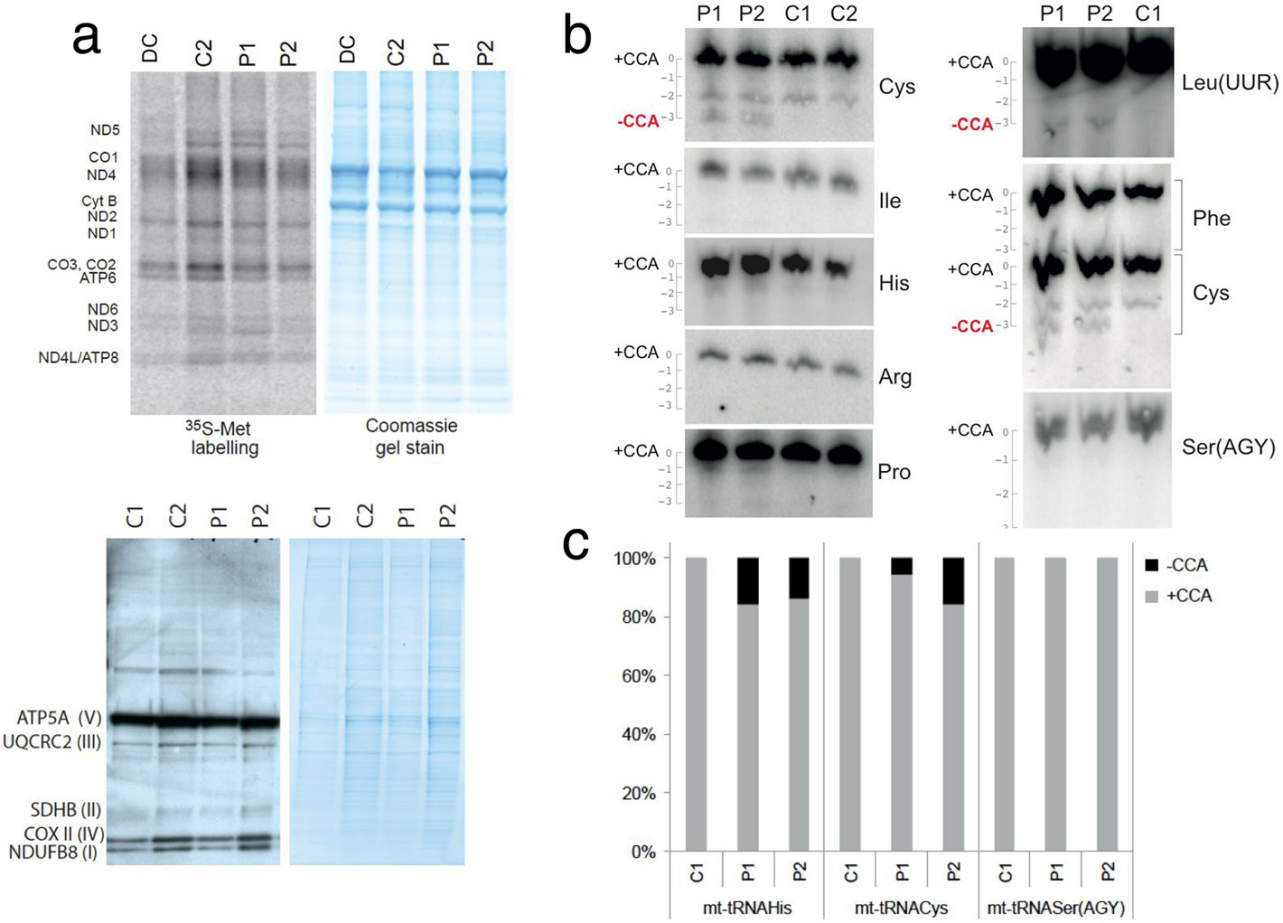
**a** Upper: Mitochondrial translation assay in disease control (DC) and patient (P1 and P2) fibroblasts. Coomassie blue (below) used as a loading control. The mitochondrial translation products are indicated on the left: four subunits of complex I (ND), one subunit of complex III (cyt b), three subunits of complex IV (CO) and two subunits of complex V (ATP). Lower: western blot for OXPHOS proteins in patients P1 and P2 compared to controls. The OXPHOS subunits detected are shown to the left with their respective complexes in parentheses. **b** High-resolution Northern blot analysis of total RNA isolated from the TRNT1 deficient patient or control primary fibroblasts. The blots were probed with the mt-tRNA-specific RNA probes as indicated. Full-length mt-tRNAs are indicated as + CCA (“0” on the scale). Mt-tRNAs lacking CCA are highlighted by “-CCA” (“-3” on the scale next to each blot). **c** The results of cRT-PCR analysis for mt-tRNA^His^, mt-tRNA^Ser^ and mt-tRNA^Cys^ in control and TRNT1 deficient patients. The following number of clones were analysed for each sample: C1: 29, P2: 27, P1: 25

The mitochondrial translation rate in the TRNT1 deficient patients (P1 and P2) was within the range detected in passage matched healthy and non-mitochondrial disease controls (Fig. 3a). We sought to determine if the identified *TRNT1* mutations result in diminished CCA addition to mitochondrial tRNAs (mt-tRNAs). To this end we performed high resolution northern blotting of total RNA isolated from P1 and P2 primary fibroblasts. This analysis suggested partial lack of CCA in mt-tRNA^Cys^ (P1: 5 %, P2: 5 %, C1: <1 %, C2: <1 %) and mt-tRNA^LeuUUR^(P1: 2 %, P2: 2 %, C1: <1 %), when compared to controls. However, we detected no obvious differences in CCA addition for other mt-tRNAs tested, including mt-tRNA^Ile^^,^^-His, -Arg, -Pro, -Phe, -Ser(AGY)^ (Fig. 3b). In order to uncover more subtle differences in maturation of the 3’ end of mt-tRNA, potentially beyond Northern blotting detection limits, we analysed the ends of mt-tRNA after circularisation, reverse transcription, PCR amplification and cloning (cRT–PCR). The cRT-PCR analysis of mttRNA^SerAGY^ and mt-tRNA^Cys^ was consistent with the high resolution northern blotting analysis where we found a proportion of incorrectly processed mt-tRNA^Cys^ (Fig. 3). Also in agreement with northern blots, we did not find any defects in maturation of mt-tRNA^SerAGY^ in this additional cRT-PCR. In addition this method also detected a proportion of incorrectly processed mt-tRNA^His^ (Fig. 3c). Taken together these data indicate that *TRNT1* mutations lead to defective CCA addition to mt-tRNAs.

### Analysis of all reported patients

#### Initial presentation

Together with the patients in this report, we analysed clinical features in 18 subjects reported to date. There appears to be a spectrum of disease ranging from a neonatal-onset disease to an adult presentation. The earliest presentation was within 3 h of birth with pallor and splenomegaly. Of the 18 cases described to date, including the 4 patients in this report, more than half (10/18, 55 %) presented in the first 2 months of life (range 1 day-19 years). The most striking initial feature (9/18, 50 %) was a systemic ‘inflammatory’ illness where an infective aetiology was not identified. The febrile illness was accompanied by gastrointestinal upset such as poor feeding, diarrhoea (8/18, 44 %) and vomiting (7/18, 39 %). In one adult case [13] a febrile illness was recorded in childhood, which was thought to be due to juvenile rheumatoid arthritis, and in one paediatric case [12] lactic acidosis and vomiting was noted but fever was not reported. The inflammatory episodes appeared to wax and wane cyclically. Other first presentations included anaemia and delayed development. The adult cases presented between the ages of 18 and 21 years with impaired vision. Table 2 demonstrates key clinical features and laboratory findings in all patients.

**Table 2:** Clinical features of TRNT1 deficiency

Clinical feature	Total	Percent
Symptom/sign		
Recurrent ‘inflammatory’ episodes	14/18	78
Developmental delay	14/18	78
Sideroblastic anaemia	13/18	72
Diarrhoea	8/18	44
Vomiting	7/18	39
Sensorineural deafness	7/18	39
Seizures	7/18	39
Retinitis pigmentosa	6/18	33
Splenomegaly	6/18	31
Ataxia	5/18	28
Brittle hair	5/18	28
Hypotonia	5/18	28
Nephrocalcinosis	5/18	28
Renal tubulopathy	4/18	22
Hepatomegaly	4/18	22
Pancreatic insufficiency	3/18	17
Villous atrophy	2/18	11
Acute encephalopathy	2/18	11
Cardiomyopathy	1/18	6
Laboratory investigations		
Low or low-normal haemoglobin	16/18	89
Microcytosis	16/18	89
B lymphopaenia/hypogammaglobulinaemia	12/18	67
Anisocytosis	9/18	50
High lactate	6/18	33
Metabolic acidosis	5/18	28
High alanine	3/18	17

### Blood and immune system

Sideroblastic anaemia (13/18, 72 %) is a very significant feature of the clinical syndrome. In the majority of cases it was observed during investigation of unexplained febrile illness. Interestingly the three adult cases had an asymptomatic microcytosis with a haemoglobin often in the lower end of the reference range (~130 g/L), contrasting with the paediatric cases who had severe anaemia (Hb range 48–82 g/L). One child appeared to have a predominantly neurological phenotype with no febrile episodes or sideroblastic anaemia [12]. Overall 16 (89 %) cases had evidence of anaemia (mild in the adult cases) with microcytosis (median Hb 72 g/L, median MCV 63.4 fl). The majority of cases (12/18, 67 %) also had B cell lymphopaenia with or without an accompanying hypogammaglobulinaemia. Only two cases had a low total white cell count.

### Retinitis pigmentosa

Of the four cases followed at our institution, one had retinal dystrophy and two other cases developed pigmentary retinopathy. Overall six cases (33 %) had pigmentary retinopathy, of which three were adults. In the adult patients this was the only discernible symptomatic feature. Although largely asymptomatic in childhood, one adult patient reported a history of night blindness as a child.

### Neurological phenotype and neuroimaging

Most patients (14/18, 78 %) had developmental delay, which was global except in one case reported to have isolated motor delay. Sensorineural deafness was reported in 7/18 (39 %) cases. Seven patients developed seizures, six patients had ataxia and five patients were hypotonic. Two of our cases (P1 and P2) developed episodes of encephalopathy. Only one patient was reported to have ptosis and ophthalmoplegia. Four patients had cerebral atrophy, two were noted to have brainstem changes, one had thalamic changes and two had cerebellar changes (atrophy in one, cerebellar hypoperfusion in the other) on neuroimaging. Two of our cases (P1 and P2) also had multiple cerebral and cerebellar focal lesions which resembled infection (Fig. 2).

### Kidney, liver and spleen involvement

Six patients (33 %) had splenomegaly. Five patients had nephrocalcinosis (28 %) and four (22 %) had hepatomegaly. One of the cases followed at our institution (P3) developed a urate renal calculus.

### Gastrointestinal

In addition to some patients developing cyclical vomiting and diarrhoea accompanying the febrile illness, three cases had pancreatic insufficiency and two had partial villous atrophy on duodenal biopsy.

### Cardiomyopathy

Only one case was reported to have (dilated) cardiomyopathy.

### Additional features

Brittle hair was noted in five cases.

### Survival

Median age of death was 37.5 months (range 10 months - 14 years). The three adult patients were alive and systemically well at 18, 19 and 21 years. Two patients [10] underwent myeloablative allogenic bone marrow transplant (BMT). Both were alive 3 years post BMT with no recurrence of periodic fever.

### Metabolic and biochemical testing

Analysis of findings in all cases revealed that an elevated lactate was recorded in six cases, associated with metabolic acidosis in five cases, elevated plasma alanine in three cases and evidence of renal tubulopathy in four cases.

We performed detailed metabolic investigations in the patients seen at our institution (Table 1). Blood lactate was mildly elevated in all four subjects (P1-P4) in the current study, ranging from 2.4–3.1 mmol/L (reference range <2). Plasma alanine was elevated in three subjects (patient range 458–875; reference range 150–450 μmol/L) and proline was elevated in two subjects (patient range 304–452; reference range 85–290 μmol/L). Plasma threonine was high in all patients (patient range 256–556; reference range 70–220 μmol/L) and plasma glycine was raised in three cases (patient range 332–504; reference range 100–330 μmol/L). Branched chain amino acids and ornithine were elevated in P3 and P4. Interestingly P4 had a normal amino acid profile post bone marrow transplant.

Electrolyte abnormalities in P1 and P3 observed during acute inflammatory episodes included hyponatraemia, hypokalaemia, hypocalcaemia, hypomagnesaemia and hypophosphataemia. Renal tubular dysfunction was documented in 3 patients (P1, P2 and P3; not done in P4), with high urinary N-acetyl-beta-D-glucosaminidase (NAG):creatinine and retinol binding protein (RBP):creatinine ratios. In P1 elevated hepatic transaminases were a persistent feature.

### Muscle respiratory chain enzymes

Muscle respiratory chain enzymes (RCEs) were measured in two of the four patients (P1 and P3) described here in detail. P3 had definitively low complex IV of 0.007, 30 % of control mean (reference range 0.014-0.034) whereas P1 had a value at the lower end of the reference range (0.015), which was disproportionately low compared to complexes I-III (Table 1). In one patient (N) reported previously complex IV was affected in muscle (0.25, control range 0.58–2.5) and liver (0.25, control range 0.6–2.5) while complex II + III 0.06 (0.5–1.9) and complex I + III 0.15 (0.58–2.5) were also low in muscle [12].

### Genotypes and protein modelling of mutations

**Fig. 4:**
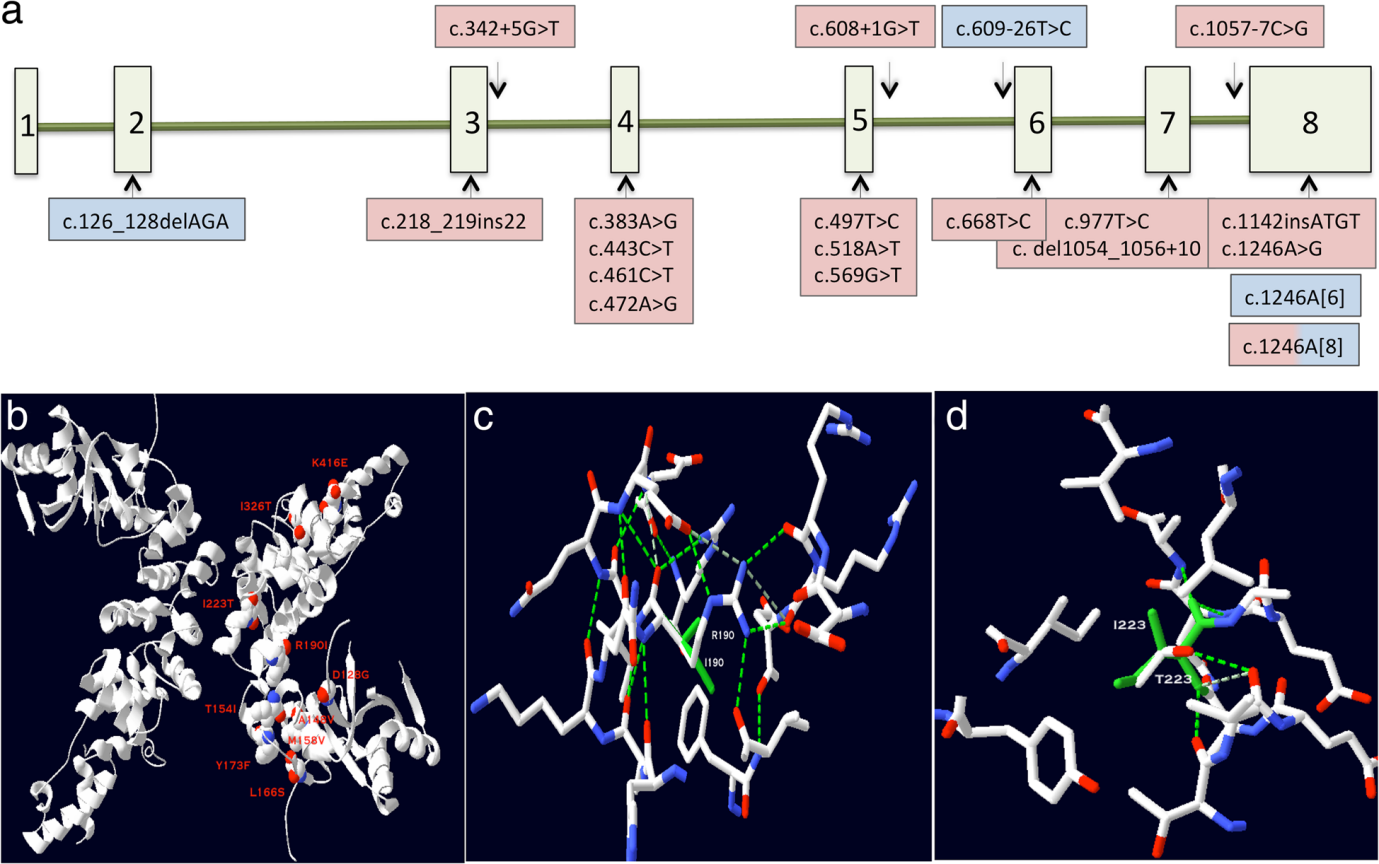
**a** Schematic diagram of the *TRNT1* gene demonstrating the mutations reported to date. Mutations found in the milder adult phenotype are shaded in blue and those found in childhood-onset severe disease are shaded in pink. **b** The human TRNT1 protein is shown in ribbon representation. All missense mutations published to date are shown as space fill atoms, white for carbon, red for oxygen and blue for nitrogen. The mutant residue is shown at each position and labelled. **c** Stick representation of the environment surrounding the p.Arg190Ile mutation. Carbon atoms in white, oxygen in red and nitrogen in blue. Ile190 is shown in green throughout. Hydrogen bonds are shown as dotted lines. The extensive hydrogen bond network stabilising the wildtype Arg190 is completely lost following mutation to the hydrophobic Ile190. **d** Stick representation of the environment surrounding the p,Ile223Thr mutation. Carbon atoms in white, oxygen in red and nitrogen in blue. Wildtype Ile223 is shown in green throughout. Hydrogen bonds are shown as dotted lines. The protein is able to accommodate the Thr223 mutation in place of the buried, hydrophobic Ile223 through the hydrogen bond from the Thr223 sidechain

All *TRNT1* mutations published to date are shown in Fig. 4a and all reported missense mutations are modelled *in silico* in Fig. 4b. The homozygous p.Arg190Ile substitution harboured by P3 and P4 is predicted to cause a severe conformational protein change when the hydrogen bond network formed by arginine is broken by isoleucine, leaving the protein either unfolded, or folded but dysfunctional (Fig. 3c).

One of the mutations found in P1 and P2 (c.688C>T, p.Ile223Thr) was also found in SIFD patients from eight apparently unrelated pedigrees of white Caucasian, Afro-Caribbean and Hispanic origins reported previously [11]. This suggests that this may be a mutational hotspot. *In silico* modelling of p.Ile223Thr showed that isoleucine is a buried amino acid and, since the substituted threonine can also be buried satisfying its H-bond, it confers a mild phenotype.

The adult patients had either a mononucleotide deletion or insertion of an adenine at the same position (c.1246) on one allele. These changes were located in the last exon and predicted to result in a truncated protein. Interestingly the deletion at c.1246 was also found in a paediatric SIFD patient [11]. The other allele in the adult patients contained either a deletion or splicing mutation; the authors predicted that the deletion caused a minor change to the protein conformation and that the splicing was altered in a subset of transcripts [13].

## Discussion

We report a new family with *TRNT1* mutations, including a novel pathogenic splicing mutation, and show that *TRNT1* mutations cause a complex multisystem disease leading to manifestations in most organs. In this report we used a systematic review of all previously reported cases to characterise the phenotypic presentation of TRNT1 deficiency, a new disorder of mitochondrial and cytoplasmic translation. Our in vitro studies provide evidence of mitochondrial pathogenicity in this disease, indicating clear functional impairment of the addition of CCA to mt-tRNAs in patient fibroblasts, leading to defective mt-tRNA post transcriptional modification.

From the descriptions thus far, there are two ends of the disease spectrum: a paediatric disorder which starts early in life with unexplained cyclical febrile episodes accompanied by gastrointestinal upset and features of sideroblastic anaemia, and an adult disease with retinitis pigmentosa and asymptomatic microcytosis [10–13]. In addition to being almost the only sign of adult disease, retinitis pigmentosa was also found in three paediatric patients, suggesting that this may be a key feature of the disease which is under-reported, since it may be undetected if patients are not specifically screened by an experienced ophthalmologist. Also splenomegaly, nephrocalcinosis and hepatomegaly were reported in six, five and four cases respectively.

We highlight oxidative phosphorylation (OXPHOS) impairment in TRNT1 deficiency, with evidence of mitochondrial complex IV being definitively low in two patients and at the lower end of the reference range in one patient. It is not possible to know how many patients would display OXPHOS abnormalities in this disease if muscle respiratory chain enzyme analyses were performed in all patients. Given the pivotal role of TRNT1 in mitochondrial translation, multiple RCE abnormalities would be expected. The observation of mtDNA translation defects with biochemical evidence of isolated complex IV deficiency is similar to other mitochondrial translation disorders (e.g., RARS2 and YARS2 deficiency) where biochemical findings ranged from normal to multiple RCE defects or isolated complex IV deficiency [20, 21]. The multi-organ involvement in these patients is perhaps unsurprising given the ubiquitous nature of the TRNT1 enzyme and its key role in protein synthesis both in cytoplasm and mitochondria. One of the limitations of this study is that we have not studied the effect of the *TRNT1* mutations on cytoplasmic tRNAs. As a dually localised enzyme, TRNT1 is responsible for the maturation of both nuclear and mitochondrially encoded tRNAs. Whilst an affect on cytosolic tRNA maturation cannot therefore be ruled out, we chose to focus on defects of the mitochondrial translation system, in view of the clinical presentation of a mitochondrial disease in our patients along with others previously reported [12]. Primarily mitochondrial disease presentations have been previously reported for other dually localised enzymes such as PUS1, MTO1 and TRIT1 [22–24], perhaps indicating a greater susceptibility of the mitochondrial translation apparatus to such perturbations than its comparatively more robust cytosolic counterpart.

Variable changes were observed in the plasma amino acid profiles of these four children with TRNT1 deficiency. It is difficult to ascertain the relevance of these findings in a small group of patients. One possible explanation is that impaired muscle metabolism leads to decreased break down of branched chain amino acids. On the other hand the abnormalities could be related to TRNT1 deficiency and resulting backlog from disordered cellular protein translation and further studies will be needed to establish this.

Often patients with inherited metabolic disease decompensate with febrile illness and metabolic physicians are involved in the care of these patients during these acute phases. In all four patients presented here, febrile illness was a component of the disease rather than a precipitant and mevalonate kinase deficiency (which is characterised by recurrent episodes of fever in infancy) was excluded. TRNT1 deficiency produces a complex multisystem disease which can be confused with overwhelming sepsis given the febrile episodes with raised inflammatory markers. Whereas sepsis obviously must be excluded in the first instance, clues at this stage are the presence of microcytic anaemia with anisocytosis and B cell lymphopaenia, typically with hypogammaglobulinaemia.

The paediatric TRNT1 disease (SIFD) enters into the differential diagnosis of genetic sideroblastic anaemia, inherited immunodeficiencies and also of autoimmune inflammatory disease. Other genetic sideroblastic anaemias which present similarly and may need exclusion include Pearson syndrome (caused by mtDNA deletions) with sideroblastic anaemia, exocrine pancreatic failure and pancytopaenia [25]; and YARS2 and PUS1 deficiency which cause myopathy, lactic acidosis and sideroblastic anaemia (MLASA) syndrome [26–28]. YARS2 is specifically a mitochondrial aminoacyl tRNA synthetase whereas PUS1 has a dual subcellular location. Of the cytoplasmic translation defects, *LARS* mutations have been noted to present with a multi-system disease consisting of acute liver failure, anaemia, renal tubulopathy, developmental delay, seizures, failure to thrive and deterioration of liver function with illness [29]. Cyclical fever and B cell immunopathy are not features of these diseases, and so they can be distinguished clinically from TRNT1 deficiency.

The mainstay of the acute management of these patients consists of treatment of the episodes of inflammation with rehydration and electrolyte supplementation and transfusion for anaemia. So far two patients have received BMT and the results appear encouraging, although more long-term data are needed. It is not known whether BMT early in the disease course might prevent the neurodevelopmental features of this disorder. Ideally randomised double-blinded placebo-controlled clinical trial evidence is necessary to draw meaningful conclusions but this is not always possible in ultra-rare diseases [30].

*TRNT1* null mutations are probably embryonically lethal given that siRNA knockdown of the gene led to cytotoxicity and apoptosis [11]. Although TRNT1 functions both in the cytoplasm and the mitochondrion, the presence of syndromic manifestations typical of a mitochondrial disease suggests that post-transcriptional modification of mt-tRNAs, specifically CCA addition, is critical to disease pathogenesis. We examined the impact of the patient mutations on tRNA modification in primary patient fibroblast cell cultures and found mt-tRNAs partially lack 3’ CCA in mt-tRNA^Cys^, mt-tRNA^LeuUUR^ and mt-tRNA^His^. While being beyond the scope of the current study, it would be interesting to study all mt-tRNAs using cRT-PCR in patients with different *TRNT1* mutations since our finding contrasts with the findings of a previous study which suggested that only mt-tRNA^Ser(AGY)^ was reduced, with more profound decrease of CCA-tailed mt-tRNA^Ser(AGY)^ in a patient with the more severe clinical phenotype [12]. We did not find a reduction in mt-tRNA^Ser(AGY)^ on Northern blot or cRT-PCR. The reasons for these differences are not clear at present but one possible explanation is that these functional differences and patient survival are related to the severity of the genetic mutation. For example it could be hypothesised that the long survival of patient 8 reported by Wiseman et al. [10] who was homozygous for p.Ile223Thr, is explained by the amino acid change where *in silico* analysis revealed a mild conformational change in the protein. In severely affected compound heterozygote patients with p.Ile223Thr in one allele, the splicing mutation found in the opposing allele may explain the severe phenotype.

## Conclusion

In summary we investigated patients with a complex, childhood-onset, multisystem disorder who fall into the SIFD spectrum of TRNT1 disease. In addition adult onset retinitis pigmentosa has been reported in TRNT1 deficiency. We demonstrate that deficiency of TRNT1 had an impact on mt-tRNA post-transcriptional modification in affected patient cells. Systematic review of all cases reported to date allowed us to amalgamate knowledge of the clinical phenotype. This will aid clinical recognition of further cases. Early bone marrow transplantation should be considered in these cases to halt the episodic febrile and metabolic decompensations, thereby improving quality of life.

## Abbreviations

BMT, bone marrow transplant; cDNA, complementary deoxynucleic acid; cRT–PCR, circularisation reverse transcription PCR; GATK, Genome Analysis Tool Kit; MRI, magnetic resonance imaging; mt-tRNA, mitochondrial transfer ribonucleic acid; OXPHOS, oxidative phosphorylation; PCR, polymerase chain reaction; RCE, respiratory chain enzymes; SIFD, sideroblastic anaemia, immunodeficiency, fevers, and developmental delay; tRNA, transfer ribonucleic acid; TRNT1, CCA-adding transfer RNA nucleotidyl transferase; WES, whole exome sequencing
